# Laser damage resistance of polystyrene opal photonic crystals

**DOI:** 10.1038/s41598-018-22831-7

**Published:** 2018-03-14

**Authors:** Lei Pan, Hongbo Xu, Ruizhen Lv, Jun Qiu, Jiupeng Zhao, Yao Li

**Affiliations:** 1 MIIT Key Laboratory of Critical Materials Technology for New Energy Conversion and Storage, School of Chemistry and Chemical Engineering, Harbin Institute of Technology, Harbin, 150001 China; 2 Center for Composite Materials and Structure, Harbin Institute of Technology, Harbin, 150001 China; 3 School of Energy Science and Engineering, Harbin Institute of Technology, Harbin, 150001 China

## Abstract

The laser damage behavior of three-dimensional photonic crystals (3D-PCs) with an opal structure is investigated using both experimental and simulation methods. Polystyrene (PS) colloidal crystals films with a reflection peak at 1064 nm are used as the model material. Disordered films (DF) are fabricated with PS microspheres as contrast samples. The laser-induced damage threshold (LIDT) of 3D-PCs is tested, which is as 2.35 times high as the LIDT of DF. All laser damages are derived from defects in 3D-PCs, implying that the LIDT of ideal 3D-PCs will be significantly increased. The simulation results show that the electric field is contained in the pores of 3D-PCs while it is reduced in the PS microspheres, which may decrease the absorption of laser energy by 3D-PCs. In contrast, the electric field distribution is irregular in DF. Enhanced electric field areas are located in both the pores and microspheres of DF. Considering higher electric field intensity causes more energy absorption and higher temperature, the DF have a lower LIDT. The 3D-PCs structure uses ordered vacancy to contain and strike back laser energy and can increase the LIDT without changing the chemical composition of materials.

## Introduction

Photonic crystals (PCs) are a class of artificial materials with photonic band gaps (PBGs) that originate from their periodic dielectric structures^1^. Many laser-related applications of PCs are being proposed or demonstrated, such as all-optical circuits^2^, omnidirectional reflectors^3^, high power laser applications^4^, high-Q nanocavity lasers^5,6^, and low-threshold lasers^7–9^, owing to its ability to manipulate light. For a versatile tool to generate and manipulate laser, laser-induced damage threshold (LIDT) of PCs is a key property that decides the whole performance^10^.

Traditional optical coatings such as high reflection coatings, anti-reflection coatings, and beam splitters are composed of periodic dielectric films and can be regarded as one-dimensional PCs (1D-PCs). The origin, nature, and characteristics of the LIDT of 1D-PCs have been extensively studied^10–17^. Contamination and structural defects give rise to most failures in 1D-PCs^10,16^ and lead to a much lower LIDT than the LIDT of bulk materials.

PBG fiber, a kind of two dimensional PCs (2D-PCs), attracted attention as candidates for the transport of very high power laser pulses recently^18^. It is reported that a PBG fiber carries most of the transmitted power in its hollow core and as little as 0.2% of the energy in the silica^19^, which results in a LIDT representing an order of 4 improvement compared to solid-core photonic crystal fibers^20^. Similar results are reported for two dimensional periodic arrays of nano-horns as anti-reflection layers^21–24^. The chalcogenide glass with 2D-PCs shows almost a factor of 2 improvement in the LIDT compared to untreated bulk glass^22^ mainly because no foreign material is introduced^21^. The strong electric field region for 1064 nm laser is distinctly distributed in the hollow part of the periodic array, which may also promote the LIDT^21^.

Three dimensional ordered^25,26^ and disordered^25,27,28^ nano-structures were also manufactured for high power laser applications. Li *et al*. observed a slightly higher LIDT in ordered porous silica coating than in the disordered one^25^. Despite the difference in absorption, the authors believe that the ordered structure is the key to interpret the high LIDT. However, *Du et al*. argued that for a small period-to-wavelength ratio, electromagnetic fields do not vary spatially and the periodicity of nanostructures would be less important to their performance^27^. In this sense, the ordered nanoporous coating was not a typical photonic crystal for the laser used in the testing. High LIDT was reported for three dimensional disordered nanoporous antireflective coatings as well and the influence of orderliness on LIDT was not discussed^27,28^.

An interesting result can be found if one compares the LIDT investigation of 1D and 2D photonic crystals, despite different testing methods, materials, and substrates, the LIDT of 1D-PCs is generally much lower than that of the substrates^10–17^ while the LIDT of 2D-PCs is close to or higher than the LIDT of substrates or bulk materials^18–24^. Many reasons may underlie this result, the ways by which the two kinds of ordered structures were constructed being one of them. 1D-PCs must consist of at least two dielectric materials (both solid usually) layer by layer and laser needs to propagate through them in sequence. The layer with the lowest LIDT, which may be defined by its chemical nature, contamination, or defects in the interface, will be the shortest stave in the barrel. However, in the 2D-PCs, the second dielectric material is usually a vacancy (air or vacuum) whose LIDT is much higher than all solid materials. Besides, the manner in which laser passes through the 2D-PCs may be different from 1D-PCs. By elaborately designing the arrangement of solid material, the bulk of laser energy can be confined within the vacancy to avoid the damage of the solid part such that the LIDT of the whole 2D-PCs is promoted. This mechanism accords with the old Chinese philosophy that vacancy makes tools more useful (Tao-te-ching, chapter 11, about 500BC).

Vacancies exist in 3D-PCs as well; however, solid materials in three dimensions baffle them periodically. Until now, the influence three-dimensional ordered structures with periods falling in the range of laser wavelength exert on the LIDT was unknown. Hereby we carried out experimental and simulative research on this problem.

## Results and Discussion

### Experiments results

**Figure 1:**
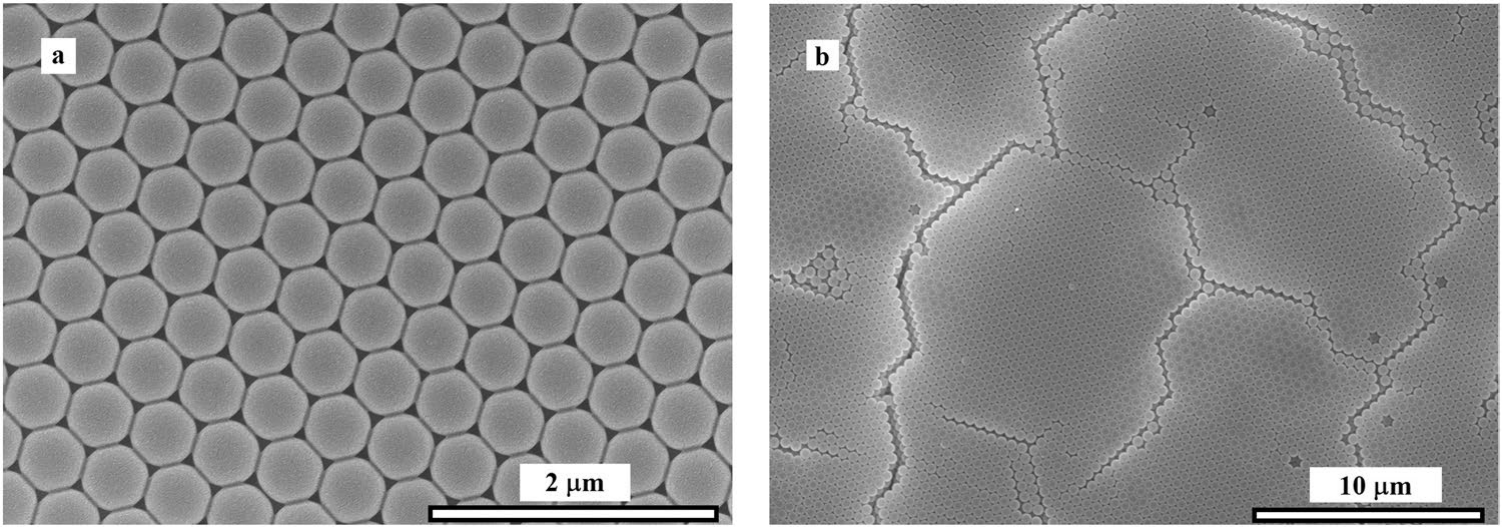
SEM images of PS 3D-PCs with a pseudo band gap at 1064 nm. (**a**) well-ordered architecture in several microns range; (**b**) defects in tens of microns range.

Among the various preparation methods for 3D-PCs, self-assembly of PS microspheres is most widely used, as it is convenient and low cost. Moreover, the low melting point of PS is in favor of the LIDT test. Hence, we choose PS 3D-PCs to conduct the laser-induced damage investigation. To format a pseudo band gap centered at 1064 nm, the average diameter of PS microspheres was carefully adjusted to 465 nm. The PDI of the diameter was 1.04. PS 3D-PCs were grown on glass slides by vertical deposition^29,30^. The SEM images of PS 3D-PCs are shown in Fig. 1. It is observed in the Fig. 1a that the deposited PS 3D-PCs have a well-ordered nano-architecture consisting of close-packed spheres. However, as shown in Fig. 1b, point defects and planar defects also exist in a larger range.

**Figure 2:**
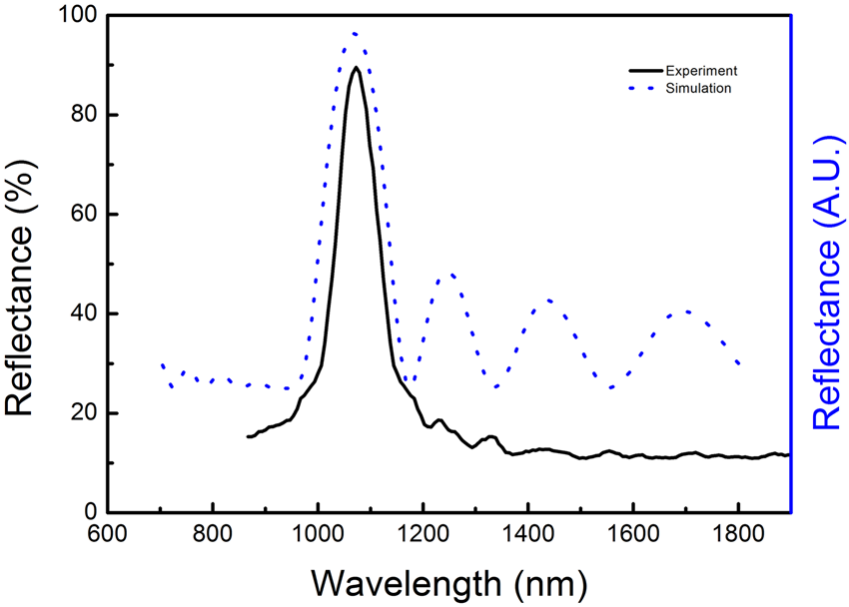
Experiment and simulation reflectance spectra of PS 3D-PCs.

The experimental reflectance spectrum of the PS 3D-PCs is given in Fig. 2. A reflective peak near 90% can be found for PS 3D-PCs in Fig. 2, whose location is very close to the wavelength of Nd:YAG laser, indicating the highly ordered structure of the PS 3D-PCs.

### LIDT test

An Nd:YAG laser with a wavelength of 1064 nm was used as laser source for the LIDT test in continuous wave mode. The 1/e^2^ spot diameters measured via the knife-edge method were 3.0 mm and the effective spot sizes were 7.1 mm^2^. The beam of laser was collimated instead of focused because the high power density is not necessary for the damage of PS.

**Figure 3:**
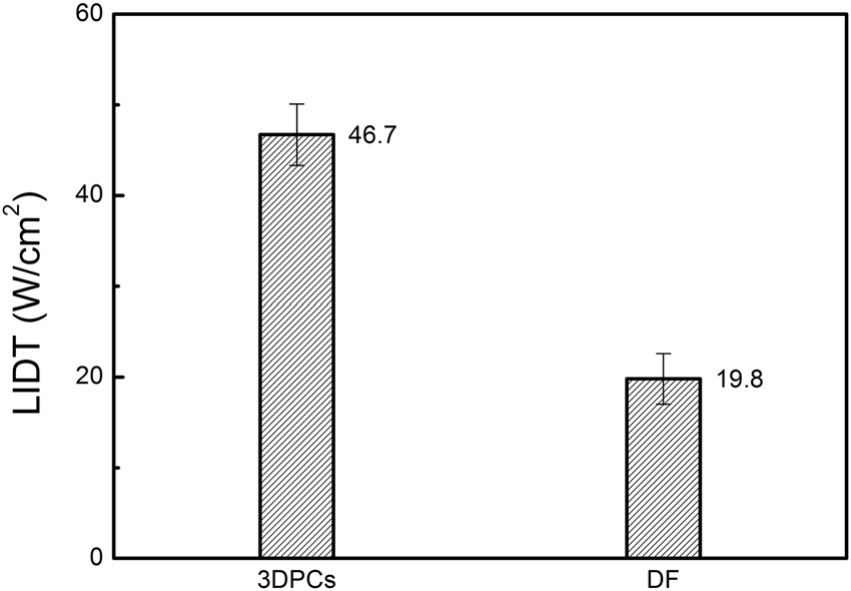
LIDT for PS 3D-PCs and DF.

The damage testing was performed as per the R-on-1 regime (i.e. multi tests per sample with increasing laser power until the occurring of damage). Ten 3D-PCs samples were irradiated and each sample was irradiated atone spot only. The damage was estimated via the visual inspection of the melting of PS membrane firstly and then confirmed by SEM examination. The LIDT of each sample was defined as the minimum laser power density for which the damage occurred within 30 s irradiation. In fact, all damage took place between 10–12 s under the irradiation of LIDT power. If damage did not occur in 30 s, it would not ensue for at least 2 min. Once the melting was detected, the laser was switched off immediately so that the initial stage of laser-induced damage can be observed subsequently. Eventually, the LIDT of 3D-PCs was obtained by averaging each sample. Moreover, to identify the influence of ordered structure on LIDT, two kinds of PS microspheres with different diameters were mixed together on purpose and disordered films (DF) were fabricated with these mixed microspheres using the vertical deposition method under the same fabrication conditions as of 3D-PCs. Ten DF were tested via the same procedures. The results of LIDT test for3D-PCs and DF are given in Fig. 3.

The test results show that the DF starts to melt under the laser illumination at 19.8 W/cm^2^ while 3D-PCs films start melting at a considerably high value, i.e., ~46.7 W/cm^2^. The LIDT of materials comprised of same PS microspheres increased for more than 135% with ordered structure. The 3D-PCs structure can realize excellent resistance to laser damage without changing the chemical component of materials.

**Figure 4:**
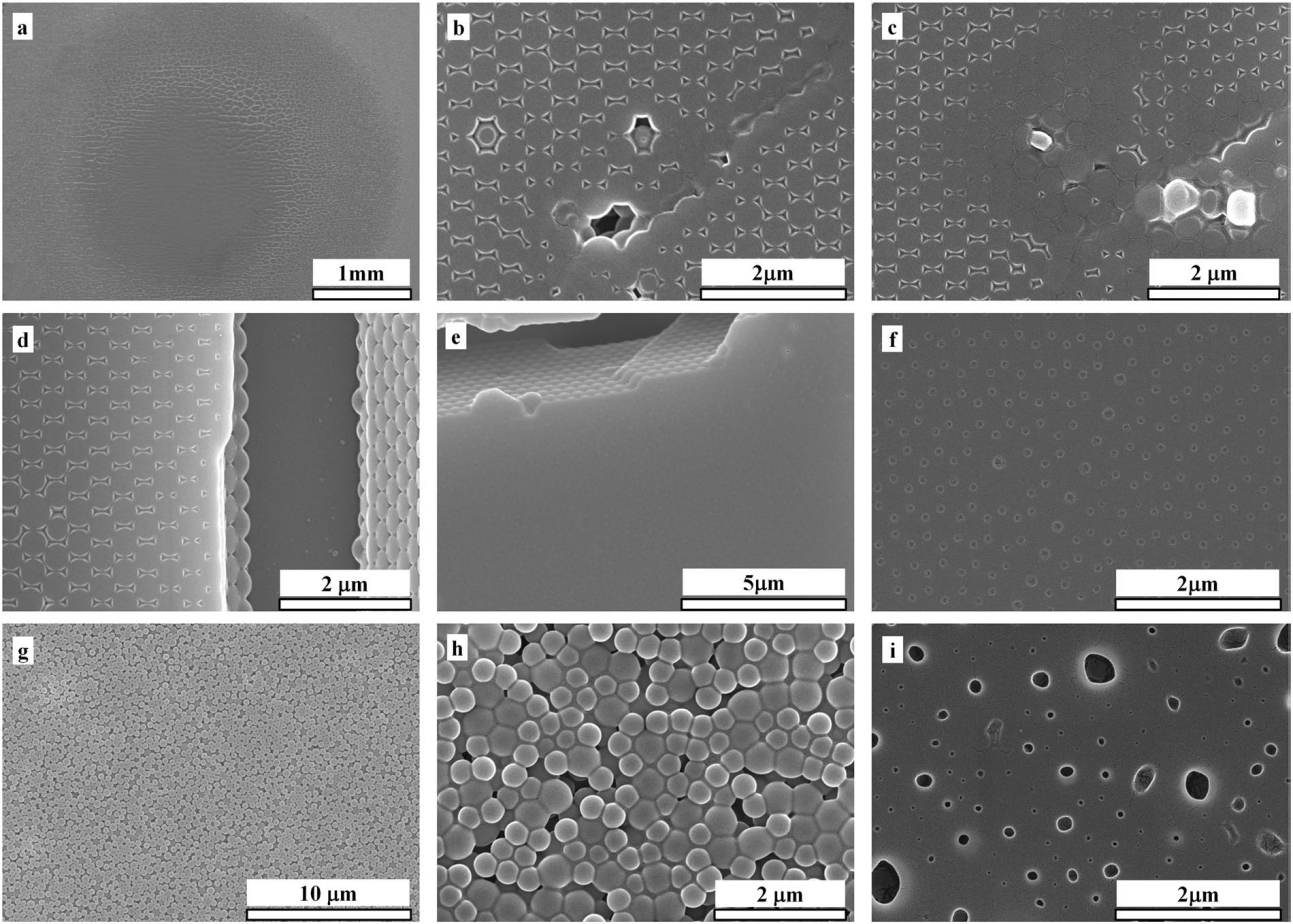
SEM images of 3D-PCs with a pseudo band gap at 1064 nm (**a–f**) and DF (**g–i**) before or after 1064 nm laser irradiation. (**a**) The whole irradiation spot on 3D-PCs, (**b–e**) different defects after irradiation, (**f**) severely melted 3D-PCs, (**g**) DF before irradiation, (**i**) lightly melted DF (**h**), severely melted DF.

The SEM images of damaged samples are given in Fig. 4. Figure 4a is the whole irradiation spot on 3D-PCs. It is observed from Fig. 4e that the melting of PS occurs first on the top layer of the photonic crystals, while the lower-layers of the photonic crystal structure are relatively well preserved. Defects such as sphere missing, surface contamination, dislocations, and cracks are inevitable in 3D-PCs prepared by vertical deposition. The illuminated defective areas are exhibited in Fig. 4b–e. As seen from these figures, more serious melting occurs at defective regions than surrounding ordered regions, which is consistent with our expectations. Defects are generally regarded as a source of electromagnetic field disturbance, causing micro-focus effect and leading to the damage of materials. The fact that laser damages of 3D-PCsare derived from defects further implies that if the defects could be effectively removed, the LIDT of perfect 3D-PCs would be significantly increased. Moreover, it can be speculated reasonably that the redistribution of electromagnetic field caused by the ordered structure of 3D-PCs neither gives rise to the harmful micro-focus in the PS microspheres lattice nor promotes the melting of PS like disordered structures. Near-field simulation will be employed to prove this speculation.The morphology of DF before irradiation is exhibited in Fig. 4g. Microspheres are piled together in a jumble as expected. Homogeneous melting occurs in the DF under laser irradiation and aggravates uniformly with the time, as noted from Fig. 4h and i.

### Simulation

Various investigations showed that the LIDT of optical materials is closely related to the electric field intensity(EFI) in the materials and the distribution of the electric field (DEF) throughout the materials is taken as an indication of susceptibility to damage^31^. This is because although the materials used in these researches are almost transparent, very small but non-negligible absorptance for laser still exists owing to impurities, which gives rise to thermal effect and is responsible for most laser induced damages. Absorption of laser is positively related to the EFI. Therefore, it is necessary to investigate the DEF in 3D-PCs for further interpreting how ordered structures affect the LIDT of materials. The DEF intensity of 3D-PCs and DF was simulated numerically using finite-difference time-domain method.

**Figure 5:**
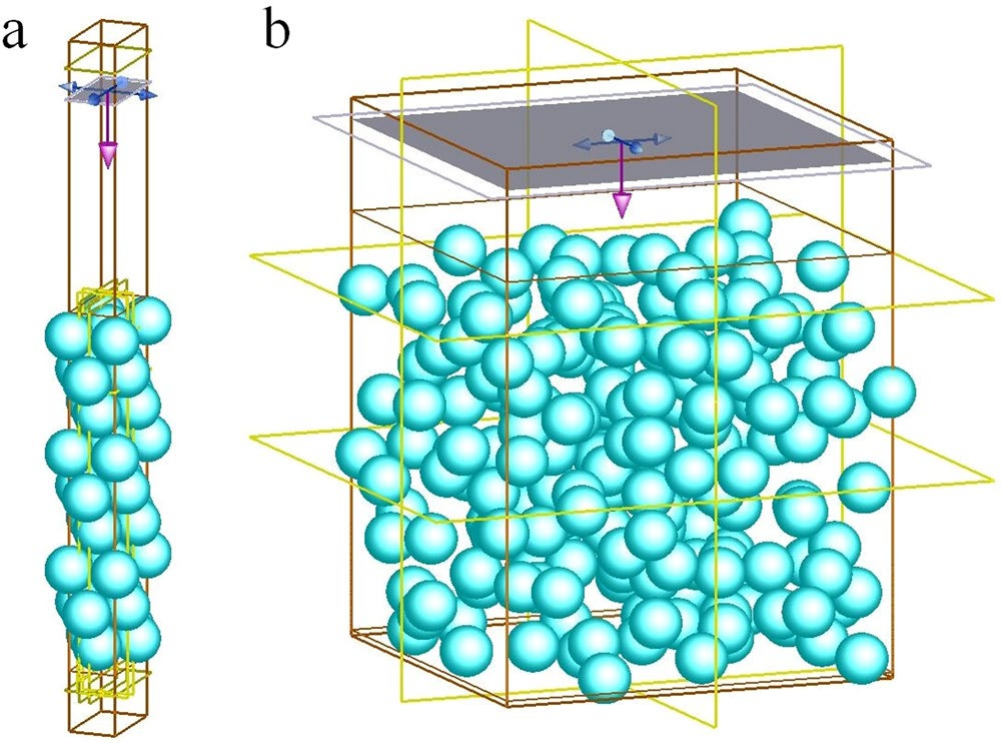
Simulation unit of (**a**) 3D-PCs and (**b**) DF. 3D-PCs consist of 9 layers of PS microspheres in opal structure. DF consist of random distributed PS microspheres. Periodic boundary conditions were set around a unit cell, while perfectly matched layer absorbing boundary conditions were used at the top and bottom boundaries of the cell.

The unit cell of both 3D-PCs and DF were designed in rectangle lattices, as illustrated in Fig. 5a and b, respectively. Periodic boundary conditions were set around a unit cell, while perfectly matched layer absorbing boundary conditions were used at the top and bottom boundaries of the cell. The incidence optical source was set as unpolarized plane waves at normal incidence.

**Figure 6:**
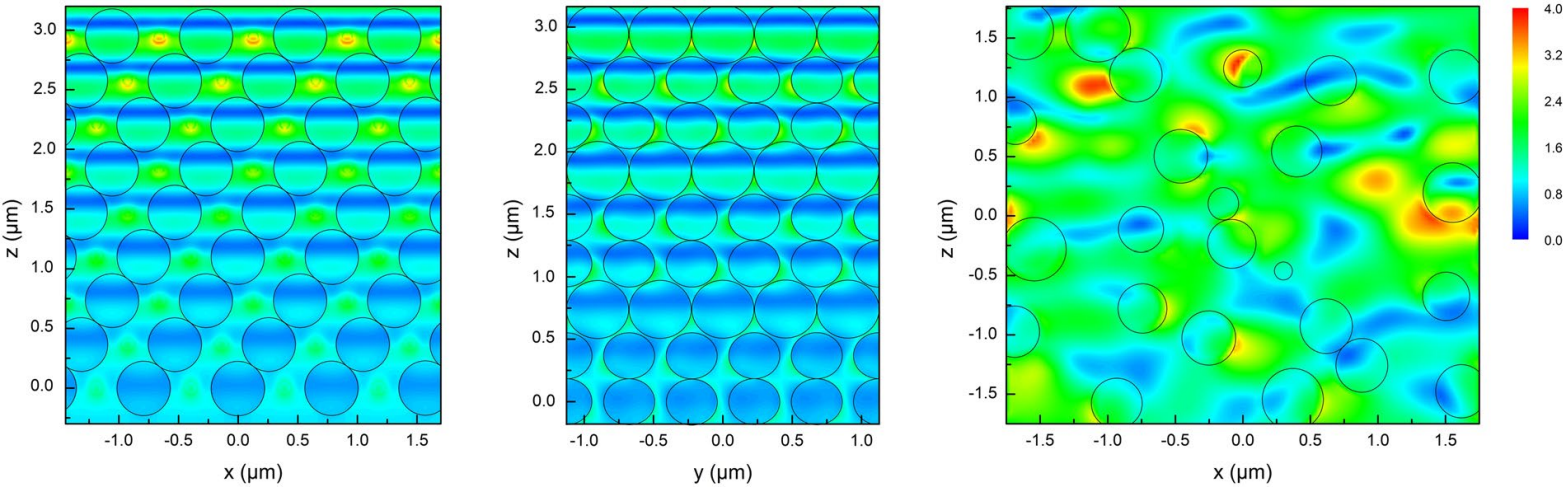
Distribution of electric field in (**a**) x-z plane of 3D-PCs, where y = 0, (**b**) y-z plane of 3D-PCs, where x = 0 and (**c**) x-z plane of DF, where y = 0.

The reflective spectrum of 3D-PCs was simulated using a source with a wavelength range from 700 nm to 1800nm and was plotted in Fig. 2. The position of the reflective peak agrees well with the experimental result. The DEF was simulated using a source with a wavelength at 1064 nm. Figure 6a and b depict the normalized DEF images of the x-z plane, where y = 0 and y-z plane, where x = 0, respectively. Black lines highlight the boundaries of PS microspheres. It is immediately noticed that the DEF in 3D-PCsis highly ordered. The EFI is mainly distributed in the vacancies among PS microspheres, and the EFI inside the PS microspheres is significantly lower than that in the vacancies, which is obviously beneficial to elevate the LIDT of the entire film. The ordered vacancies firstly contain the energy of laser without damaging the material and then reflect it backwards. This principle is similar to the traditional Shadow Boxing conceived from Tao-te-ching, which fights back the strongest strike with nothing. Besides, it can be seen that the EFI is rapidly attenuated due to the pseudo forbidden band in the z-direction; the EFI in the upper most level, both inside and outside the microspheres, is the highest, which is consistent with the results that the first-level microspheres melt the most, as shown in Fig. 4e.

On the contrary, the DEF of disordered PS microsphere films is very disordered and nonuniform, as illustrated in Fig. 6c. Owing to the “micro-focus” effect of disordered structures, the highest EFI is almost 4 times that of the source. In addition, the “micro-focuses” locate randomly both in vacancies and PS microspheres. The average EFI inside the microspheres of disordered PS films is significantly higher than that of 3D-PCs, which naturally leads to higher absorption and lower LIDT. Besides, laser propagates through the disordered PS film without evident attenuation.

## Methods

All reagents except water used were commercial Sigma-Aldrich products. High-purity water with a resistivity of 18.2 MΩ·cm at 25 °C was prepared by a water purification system (CSR-1-30T).

Monodisperse polystyrene (PS) microspheres were synthesized by an emulsifier-free emulsion polymerization technique^32^. Scanning electron microscopy (SEM) images were obtained using a Hitachi SU 8000 microscope operated at 15 kV. The average diameter of the PS microspheres was calculated from more than 100 microspheres. Polydispersity index (PDI) = D_m_/D_n−1_,where D_m_ and D_n_ were the weight-average and number-average diameters, respectively^33^. The normal-incident reflectance spectra of 3D-PCswere obtained by using an Ocean Optics NIR Quest instrument equipped with a reflection probe. A specular reflectance standard(PN A338-MS-1) with a reflectance of 85–90% was used as measured reference.

An Nd:YAG laser was used as a laser source for LIDT test on continuous wave mode. The 1/e^2^ spot diameters measured via the knife-edge method were 3 mm and the effective spot sizes were 7 mm^2^.

## Conclusions

PS 3D-PCs with a pseudo forbidden band centered at 1064 nm were prepared by vertical deposition. The LIDT of 3D-PCs is 2.35 times high as the LIDT of DF fabricated with the same material. The laser damages of 3D-PCsare derived from defects. The simulation results show that the ordered structure of the 3D-PCs plays a very interesting role of modulating the DEF of laser. The EFI is mainly contained in the vacancies among microspheres during its short propagation in 3D-PCs and the EFI inside the microspheres is significantly lower than that outside. Besides, the EFI rapidly decreases with the increase of penetration depth in 3D-PCs. Such a DEF is very conducive to preventing laser damage. In contrast, “micro-focuses” with high EFI locate randomly both in vacancies and PS microspheres in DF. This explains the higher LIDT of 3D-PCs than DF. The laser damages observed in the experiment are derived from a variety of defects. Therefore, it can be reasonably speculated that ideal flawless opal photonic crystals will have a much higher LIDT.

In short, 3D-PCs structure uses ordered vacancy to contain and strike back laser energy and can increase the LIDT without changing the chemical composition of materials.
